# Are transgender people satisfied with their lives?

**DOI:** 10.1186/s12889-023-15831-4

**Published:** 2023-05-30

**Authors:** Katharina Grupp, Marco Blessmann, Hans-Helmut König, André Hajek

**Affiliations:** 1 Division of Plastic, Reconstructive and Aesthetic Surgery, University Medical Center Hamburg-Eppendorf, Martinistr. 52, 20246 Hamburg, Germany; 2 Department of Health Economics and Health Services Research, University Medical Center Hamburg-Eppendorf, Hamburg Center for Health Economics, Martinistr. 52, 20246 Hamburg, Germany

**Keywords:** Life satisfaction, Happiness, Subjective well-being, Transgender people

## Abstract

**Background:**

Our goal was to examine the proportion of transgender people satisfied with their lives (i.e., cognitive evaluation of life as a whole) and the determinants of life satisfaction level among transgender individuals.

**Methods:**

Data were taken from the HH-TPCHIGV study. Included were 104 transgender people who had joined self-help groups to get and share information about the gender-affirming surgeries performed at the Division of Plastic, Reconstructive and Aesthetic Surgery at the University Medical Center Hamburg-Eppendorf. The established Satisfaction with Life Scale was used to quantify life satisfaction. Sociodemographic-, lifestyle-related and health-related determinants were included in multiple linear regressions. In regression analysis, life satisfaction served as outcome measure and in a robustness check ordered probit regressions were used.

**Results:**

Among transgender people, 12.9% can be classified as “extremely dissatisfied”, 18.3% can be classified as “dissatisfied”, 12.9% can be classified as “slightly dissatisfied”, 7.5% as “neutral”, 30.1% as “slightly satisfied”, 17.2% as “satisfied” and 1.1% as “extremely satisfied”. Higher levels of life satisfaction were associated with higher age (β = .15, *p* < .05), higher school education (β = 5.54, *p* < .001), and favorable self-rated health (β = 2.20, *p* < .001).

**Conclusions:**

Nearly half of the transgender people were at least “satisfied” with their lives. Knowledge about the correlates of life satisfaction may assist in addressing unsatisfied individuals.

**Supplementary Information:**

The online version contains supplementary material available at 10.1186/s12889-023-15831-4.

## Introduction

Cisgender comprise the social majority, whose gender identities or expressions are matching with their sex assigned at birth [1]. Transgender people are a gender minority, who are gender incongruent, with their identities or expressions of gender not matching the sex they were assigned at birth [1].

According to minority social stress theory, people with disadvantaged social status are more likely to be exposed to stressors and to be more vulnerable to stress because they have limited psychosocial coping resources; resulting in increased risk for negative health outcome [2].

There are substantial indications that many transgender people struggle with psychosocial issues. Recently, Eisenberg et al. [3] analyzed more than 80.000 transgender students and demonstrated that gender incongruent individuals reported higher rates of life time suicidal ideation and life time suicide attempts (about 61% and 31%) than their cisgender peers (about 20% and 7%). Moreover, high prevalence of depression has been described. For example, two studies analysing transgender women showed that prevalence was 64% in 573 [4] and 63% in 230 individuals [5].

However, little is known about the association between the level of life satisfaction of transgender people and socio-economic, lifestyle-related and health-related determinants. Most of the studies analysing satisfaction of transgender focusing on the level of surgical satisfaction or sexual well-being following surgical treatment [6–29]. Moreover, understanding risk factors for reduced life satisfaction is critical to better understand the gender needs (requirements of individuals) to improve their position or status. Addressing these needs allow people to have control over their lives beyond socially-defined restrictive roles. Thus, we aim to improve the people’s health and wellbeing, yet remarkably few studies have done so in transgender people.

## Methods

### Sample

The "Transgender Survey" HH-TPCHIGV was developed in collaboration with the Division of Plastic, Reconstructive and Aesthetic Surgery and the Department of Health Economics and Health Services Research (both at the University Medical Center Hamburg-Eppendorf, UKE, Hamburg, Germany). Data from this study were used for this present study.

Adult transgender people who had obtained information about trans-specific surgery at the Division of Plastic, Reconstructive and Aesthetic Surgery at UKE were surveyed. These transgender individuals often had registered in support groups on Facebook and Whatsapp, among other things. Included were transgender people before and after gender affirmation surgery at the Division of Plastic, Reconstructive and Aesthetic Surgery at the University Medical Center Hamburg-Eppendorf (as well as transgender people who are only in the support groups and had contact with other transgender people here who have had counseling with the Division of Plastic, Reconstructive and Aesthetic Surgery or have undergone surgery there). There were no specific exclusion criteria, such as specific chronic conditions. We used the online survey application "Limesurvey" to program, host, and conduct the survey. The data was collected between April and October 2022. Informed consent was obtained prior to participation by all individuals.

### Dependent variables

The valid and widely used Satisfaction with Life Scale (SWLS) was used to measure life satisfaction. This tool was developed by Diener et al. [30]. The SWLS consists of five items. The final score is expressed by the sum of all five items (ranging from 5 to 35), whereby higher scores reflect higher satisfaction with life. Cronbach’s alpha was 0.86 (McDonald’s omega was 0.87) in this study.

Following the recommendations by Pavot and Diener [31], it was first categorized as follows: 5 to 9 “extremely dissatisfied”, 10 to 14 “dissatisfied”, 15 to 19 “slightly dissatisfied”, 20 “neutral”, 21 to 25 “slightly satisfied”, 26 to 30 “satisfied”, and 31 to 35 “extremely satisfied”. For reasons of readability, we used a cut-off of 21 and higher to classify individuals as “satisfied” for descriptive purposes.

### Determinants

Several (i) sociodemographic, (ii) lifestyle-related and (iii) health-related determinants were selected – guided by former research in this area [32, 33]: Age, school education (with these categories: general or subject-specific university entrance qualification (e.g. “Abitur”); completion of polytechnic secondary school; currently in school education; secondary school diploma; intermediate school leaving certificate (e.g. “Realschulabschluss”); without general school leaving certificate), family life (single; divorced; widowed; living separately: married or in partnership; living together: married or in partnership), migration background (no; yes), employment situation (with these categories: full-time employed; unemployed; part-time employed; marginally employed (450-euro job or mini-job); retired/early retirement; other; in retraining; in vocational training/apprenticeship), religious affiliation (Non-denominational; Islam; Christianity; Buddhism; Other), gender affirming surgery (no; yes, already), frequency of sports activities (no sports activity; less than one hour a week; regularly, 1–2 h a week; regularly 3–4 h a week; regularly, more than 4 h a week), self-rated health (using a single-item from 1 = very bad to 5 = very good), and having one or more chronic illnesses (no; yes).

### Statistical analysis

The proportion of individuals satisfied with their lives (i.e., SWLS ≥ 21) was first shown—for the total sample and additionally for key subgroups. Moreover, the sample characteristics stratified by satisfaction with life were displayed. Thereafter, multiple linear regressions were used to explore the determinants of the level of life satisfaction. In a robustness check, ordered probit regressions were used. To calculate McDonald’s omega, we used a recently developed tool [34]. To tackle missing data, a full-information maximum likelihood (FIML) approach [35] was used in this study. The statistical significance was defined as *p* value of ≤ 0.05. Stata 16.1 was used for statistical analyses (Stata Corp., College Station, Texas).

## Results

### Sample characteristics and proportion of satisfied transgender people

The mean age was 30.4 years (19 to 63 years, SD: 9.6 years) in our current sample. In Table 1, the proportion of individuals satisfied with their lives are shown (also for subgroups). In total, about 48.4% of the transgender people were satisfied with their lives. The proportion of satisfied transgender people markedly ranged between the subgroups. For instance, 18.2% of the individuals with a migration background were satisfied with their lives, whereas 74.4% of the individuals with a general or subject-specific university entrance qualification were satisfied with their lives.

**Table 1:** Proportion of transgender individuals satisfied with their lives among several groups

	n	Satisfied	*p*-value
Total sample	*N* = 93	48.4%	
Age bracket			.99
18 to 29 years	*N* = 53	47.2%	
30 years and older	*N* = 38	47.4%	
Family situation			.18
Living separately: married or in partnership; divorced; single; widowed	*N* = 49	40.8%	
Married or in partnership	*N* = 42	54.8%	
School education			< .001
Absence of general or subject-specific university entrance qualification	*N* = 52	26.9%	
Presence of general or subject-specific university entrance qualification	*N* = 39	74.4%	
Migration background			.04
No	*N* = 80	51.2%	
Yes	*N* = 11	18.2%	
Employment situation			.08
Unemployed	*N* = 16	25.0%	
Full-time employed	*N* = 34	58.8%	
Other	*N* = 41	46.3%	
Having a religious affiliation			.41
Non-denominational	*N* = 53	50.9%	
Having a religious affiliation	*N* = 38	42.1%	
Already having a gender affirming surgery			.23
No	*N* = 50	39.6%	
Yes	*N* = 38	52.6%	
Chronic diseases			< .01
Absence of at least one chronic disease	*N* = 52	65.2%	
Presence of at least one chronic disease	*N* = 50	31.9%	

Notes: Chi^2^ tests were performed (p-values). Satisfied: Score of 21 and higher on the SWLS

A Chi^2^ test showed that being satisfied with one’s life was significantly associated with school education (*p* < 0.001). Moreover, being satisfied with one’s life was significantly associated with migration background (*p* = 0.04). Additionally, being satisfied with one’s life was significantly associated with chronic conditions (*p* < 0.01). More details are shown in Table 1. Sample characteristics stratified by satisfaction with life are shown in Supplementary Table 1.

Some more details are also worth noting regarding life satisfaction: among transgender people 12.9% can be classified as “extremely dissatisfied”, 18.3% as “dissatisfied”, 12.9% as “slightly dissatisfied”, 7.5% as “neutral”, 30.1% as “slightly satisfied”, 17.2% as “satisfied” and 1.1% as “extremely satisfied”. The average SWLS score equaled 18.7 (SD: 6.8, ranging from 5 to 30.5).

### Regression analysis

In Table 2, the findings of multiple linear regressions with the level of life satisfaction as outcome are presented. R^2^ was 0.51. Higher levels of life satisfaction were associated with higher age (β = 0.15, *p* < 0.05), higher school education (β = 5.54, *p* < 0.001), and favorable self-rated health (β = 2.20, *p* < 0.001), whereas the outcome was not associated with the other independent variables.

**Table 2:** Determinants of life satisfaction. Findings of multiple linear regressions

Independent variables	Level of life satisfaction
Age (in years)	0.15*
	(0.06)
Family situation:—Living together: Married or in partnership (Reference category: Other including [living separately: married or in partnership; divorced; single; widowed])	1.61
	(1.09)
School education:—General or subject-specific university entrance qualification (e.g. “Abitur”) (Reference: Lower school education including [Completion of polytechnic secondary school; Currently in school education; Secondary school diploma; Intermediate school leaving certificate (e.g. “Realschulabschluss”); Without general school leaving certificate])	5.54***
	(1.12)
Employment situation:—Full-time employed (Reference category: Unemployed)	1.43
	(1.49)
- Other including [Part-time employed; Marginally employed (450-euro job or mini-job); Retired/early retirement; Other; In retraining; In vocational training/apprenticeship	2.16
	(1.36)
Migration background: Yes (Reference category: No)	-0.80
	(1.67)
Religious affiliation: Having a religious affiliation including [Buddhism; Christianity; Islam; Other] (Reference category: Non-denominational)	-0.55
	(1.02)
Already having a gender affirming surgery:—Yes (Reference category: No)	-0.88
	(1.08)
Frequency of sports activities:—Less than one hour a week (Reference category: No sports activity)	-2.32 +
	(1.31)
- Regularly, 1–2 h a week	-0.78
	(1.57)
- Regularly, 3–4 h a week	1.29
	(1.57)
- Regularly, more than 4 h a week	1.04
	(1.69)
Self-rated health (from 1 = very bad to 5 = very good)	2.20***
	(0.63)
Having at least one chronic disease: Yes (Reference category: No)	-1.20
	(1.25)
Constant	3.01
	(2.94)
R^2^	0.51
Observations	104

Notes: *** *p* < 0.001, ** *p* < 0.01, * *p* < 0.05, + *p* < 0.10; Unstandardized beta-coefficients are displayed; robust standard errors in parentheses; FIML was used to handle missing values

To test the robustness of our results, we replaced multiple linear regressions by ordered probit regressions (Table 3). In terms of significance, the results remained virtually the same. Detailed results are shown in Table 3.

**Table 3:** Determinants of life satisfaction. Findings of multiple ordered probit regressions

Independent variables	Level of life satisfaction
Age (in years)	0.03*
	(0.01)
Family situation:—Living together: Married or in partnership (Reference category: Other including [living separately: married or in partnership; divorced; single; widowed])	0.23
	(0.24)
School education:—General or subject-specific university entrance qualification (e.g. “Abitur”) (Reference: Lower school education including [Completion of polytechnic secondary school; Currently in school education; Secondary school diploma; Intermediate school leaving certificate (e.g. “Realschulabschluss”); Without general school leaving certificate])	1.19***
	(0.25)
Employment situation:—Full-time employed (Reference category: Unemployed)	0.29
	(0.30)
- Other including [Part-time employed; Marginally employed (450-euro job or mini-job); Retired/early retirement; Other; In retraining; In vocational training/apprenticeship	0.54 +
	(0.30)
Migration background: Yes (Reference category: No)	-0.07
	(0.36)
Religious affiliation: Having a religious affiliation including [Buddhism; Christianity; Islam; Other] (Reference category: Non-denominational)	-0.17
	(0.23)
Already having a gender affirming surgery:—Yes (Reference category: No)	-0.24
	(0.23)
Frequency of sports activities:—Less than one hour a week (Reference category: No sports activity)	-0.49 +
	(0.28)
- Regularly, 1–2 h a week	-0.18
	(0.33)
- Regularly, 3–4 h a week	0.34
	(0.35)
- Regularly, more than 4 h a week	0.13
	(0.35)
Self-rated health (from 1 = very bad to 5 = very good)	0.52**
	(0.16)
Having at least one chronic disease: Yes (Reference category: No)	-0.38
	(0.28)
Pseudo R^2^	0.14
Observations	86

Notes: *** *p* < 0.001, ** *p* < 0.01, * *p* < 0.05, + *p* < 0.10; Coefficients were reported (larger values correspond to “higher” outcomes); standard errors in parentheses; Listwise deletion was used to handle missing values

## Discussion

Our goal was to examine the proportion of satisfied individuals and the determinants of the level of life satisfaction among transgender people.

Our data showed that a high percentage of transgender people were dissatisfied with their lives. This result is generally in accordance with the study of Anderssen et al. [36] demonstrating that transgender people reported significantly lower life satisfaction than did cisgender people. In detail, the authors showed that 70% of binary transgender and 64% of non-binary transgender people reported being dissatisfied with their lives, compared to 34–35% among cisgender people [36]. Moreover, our data are in line with the study of Chen et al. [37], analyzing 95 youth showing that life satisfaction was lower amongst transgender people as compared to population.

Regarding the impact of gender-affirming-surgery and/or hormonal therapy on satisfaction, we found discrepant data in literature. While some authors described that transgender people were more satisfied following hormonal treatment and/or gender-affirming-surgery [16, 37–46], others described that transgender women following surgery were generally dissatisfied [47]. For example, Weigert et al. [16] investigated the effects of augmentation mammoplasty on psychosocial well-being and described that transgender women reported improvements of their psychosocial wellbeing from pre- to post-operative [16]. Additionally, high levels of life satisfaction after vaginoplasty in most transgender women [42, 43] and after facial feminization surgery [44] have been detected. Contrary to these results, Meister et al. [47] described that transgender women reported that they were generally dissatisfied with their relationships with their friends, acquaintances, and relatives after undergoing vocal cord surgery [47]. In our study, the level of life satisfaction in transgender people was not increased in transgender who had undergone gender-affirming surgery as compared to those who were unoperated. A possible explanation may be that gender-affirming surgery – similar to other critical life events [48] —only had a short-term effect on life satisfaction and bounces back to the initial set point of life satisfaction after some years [49].

Interestingly, in our study, higher levels of life satisfaction were associated with higher age. Previously, authors described that transgender youth was associated with disproportionally high rates of depression, anxiety, suicidality, and non-suicidal self-injury [39, 50–52]. Such factors are known to be associated with lower life satisfaction [53–55]. Thus, they may explain why higher age was associated with higher levels of life satisfaction in our study.

Moreover, we showed that increased levels of life satisfaction were associated with higher school education. To the best of our knowledge, this is the first study demonstrating such an association. Regarding the association between school education and negative health outcome such as suicidal ideation, Pen et al. [56] demonstrated that transgender with only junior school education or below were more likely to report suicidal ideation in comparison to those who attained a college degree or higher. Individuals with higher school education may have better coping skills to deal with negative experiences compared to individuals with lower school education [57]. This may assist in maintaining satisfaction with life.

A further investigation was to determine the level of satisfaction with life and self-rated health. Here, we showed that increased levels of life satisfaction were associated with favorable self-rated health. Similarly, based on large data from a longitudinal survey taking place in Australia, Siahpush et al. [58] showed that there was a positive association between favorable self-rated health and higher levels of life satisfaction. Our study thus confirms the well-established association between self-rated health and life satisfaction among transgender individuals.

One important aspect of the life satisfaction is also sexual life. In literature, several studies analyzed the sexual wellbeing after surgery. Weigert et al [16]. investigated the longer-term effects of augmentation mammoplasty on psychosocial wellbeing and sexual wellbeing and reported that transgender women reported improvements in both their psychosocial and sexual wellbeing. Regarding the sexual wellbeing after genital surgery several studies suggest that transgender men experience positive sexual wellbeing outcomes after undergoing genital surgery. Seven studies [10, 13, 14, 17–20] reported that transgender male participant samples were satisfied with their sexual functioning, while some found that no participant reported difficulties engaging in sexual intercourse [21], while others [11, 22] reported relatively low rates of satisfaction with sexual functioning post genital surgery. Regarding transgender women, several studies analyzed the sexual wellbeing postoperative. Many studies demonstrated that transgender women reported good sexual functioning postoperatively [23–26] and found that transgender women reported high satisfaction with their sex lives overall. Moreover, several studies showed that most transgender women were able to orgasm post-surgery [19, 24, 27–29].

We would like to draw attention to some strengths and shortcomings of our study. This study determines life satisfaction exclusively among transgender people. It is important to pay attention to this because it is a vulnerable group that is difficult to reach. In addition, a very established tool was used to identify life satisfaction. The cross-sectional methodology of our study and the unclear generalizability of this hospital-based population are notable drawbacks. A further limitation is the small sample size of our study. Moreover, a shortcoming of our study is the sole focus on life satisfaction. Future studies are needed to clarify the determinants of affective well-being (such as negative or positive affect) among transgender people. Furthermore, upcoming studies are needed to investigate the determinants of sexual satisfaction among transgender people.

## Conclusion

Nealy haft of the transgender people were at least “satisfied” with their lives and higher levels of life satisfaction were associated with higher age, higher school education, and favorable self-rated health. Knowledge about the correlates of life satisfaction may assist in addressing unsatisfied individuals.

## Supplementary Information

**Additional file 1.**

## Data Availability

The datasets analyzed during the current study are not publicly available due to ethical restrictions involving patient data but are available from the corresponding author on reasonable request.
