# Supplementary material for: Are transgender people satisfied with their lives?
Source: BMC Public Health. 2023 May 30;23:1002. doi: 10.1186/s12889-023-15831-4 (PMC10227974; doi:10.1186/s12889-023-15831-4)
Supplement: Supplementary file 1 — Additional file 1. [file 12889_2023_15831_MOESM1_ESM.docx]

Supplementary Table 1. Characteristics of transgender individuals (stratified by satisfaction with life)

|  | Unsatisfied with their lives | Satisfied with their lives |
| --- | --- | --- |
|  | Mean (SD) / n (%) | Mean (SD) / n (%) |
| Age (in years) | 29.9 (8.8) | 31.0 (10.5) |
| Family situation |  |  |
| Living separately: married or in partnership; divorced; single; widowed | 29 (60.4%) | 20 (46.5%) |
| Married or in partnership | 19 (39.6%) | 23 (53.5%) |
| School education |  |  |
| Absence of general or subject-specific university entrance qualification | 38 (79.2%) | 14 (32.6%) |
| Presence of general or subject-specific university entrance qualification | 10 (20.8%) | 29 (67.4%) |
| Employment situation |  |  |
| Unemployed | 12 (25.0%) | 4 (9.3%) |
| Full-time employed | 14 (29.2%) | 20 (46.5%) |
| Other | 22 (45.8%) | 19 (44.2%) |
| Migration background |  |  |
| No | 39 (81.3%) | 41 (95.3%) |
| Yes | 9 (18.8%) | 2 (4.7%) |
| Having a religious affiliation |  |  |
| Non-denominational | 26 (54.2%) | 27 (62.8%) |
| Having a religious affiliation | 22 (45.8%) | 16 (37.2%) |
| Already having a gender affirming surgery |  |  |
| No | 29 (61.7%) | 19 (48.7%) |
| Yes | 18 (38.3%) | 20 (51.3%) |
| Frequency of sports activities |  |  |
| No sports activity | 11 (22.9%) | 9 (20.9%) |
| Less than one hour a week | 15 (31.3%) | 6 (14.0%) |
| Regularly, 1-2 hours a week | 8 (16.7%) | 7 (16.3%) |
| Regularly, 3-4 hours a week | 6 (12.5%) | 13 (30.2%) |
| Regularly, more than 4 hours a week | 8 (16.7%) | 8 (18.6%) |
| Self-rated health (from 1 = very bad to 5 = very good) | 3.3 (0.9) | 4.0 (0.7) |
| Having at least one chronic disease |  |  |
| No | 16 (33.3%) | 30 (66.7%) |
| Yes | 32 (66.7%) | 15 (33.3%) |

Notes: High satisfaction with life: Score of 21 and higher on the SWLS (low satisfaction with life otherwise)
